# Surface Deformation Analysis of the Wider Zagreb Area (Croatia) with Focus on the Kašina Fault, Investigated with Small Baseline InSAR Observations

**DOI:** 10.3390/s19224857

**Published:** 2019-11-07

**Authors:** Marin Govorčin, Boško Pribičević, Shimon Wdowinski

**Affiliations:** 1 Faculty of Geodesy, University of Zagreb, Kačićeva 26, HR-10000 Zagreb, Croatia; bpribic@geof.hr; 2 Department of Earth and Environment, Florida International University, 11200 SW 8th Street, Miami, FL 33199, USA; swdowins@fiu.edu

**Keywords:** InSAR, SBAS, StaMPS, interseismic strain accumulation, surface deformation analysis

## Abstract

The wider Zagreb area is considered one of the few seismically active areas in the Republic of Croatia. During the period 1880–1906, moderate to strong seismic activity with three earthquakes magnitude M_*L*_ ≥ 6 occurred on the NW-SE striking Kašina Fault and since then, the area has not experienced earthquakes exceeding magnitude M_*L*_ = 5. In order to estimate the ongoing interseismic strain accumulation along the fault, we analyze Advanced Land Observing Satellite (ALOS) Phased Array L-band SAR (PALSAR) and Environmental Satellite (Envisat)-Advanced Synthetic Aperture Radar (ASAR) datasets acquired over the period 2007–2010 and 2002–2010, respectively. The data were analyzed using small baseline interferometry (SBI) technique and indicate very slow surface deformations in the area, within ±3.5 mm/year, which are in a good agreement with previous geodetic studies. Interseismic strain accumulation analysis was conducted on two 14 km long segments of the Kašina Fault, seismically active in the South and stable in the North. The analysis indicates an ongoing interseismic strain accumulation of 2.3 mm/year on the Southern segment and no detectable strain accumulation on the Northern segment. Taking into consideration the lack of moderate to strong seismic activity in the past 113 years combined with the preliminary geodetic analysis from this study, we can conclude that the Southern segment of the Kašina Fault has the potential to generate earthquake magnitude M_*w*_ < 6.

## 1. Introduction

**Figure 1:**
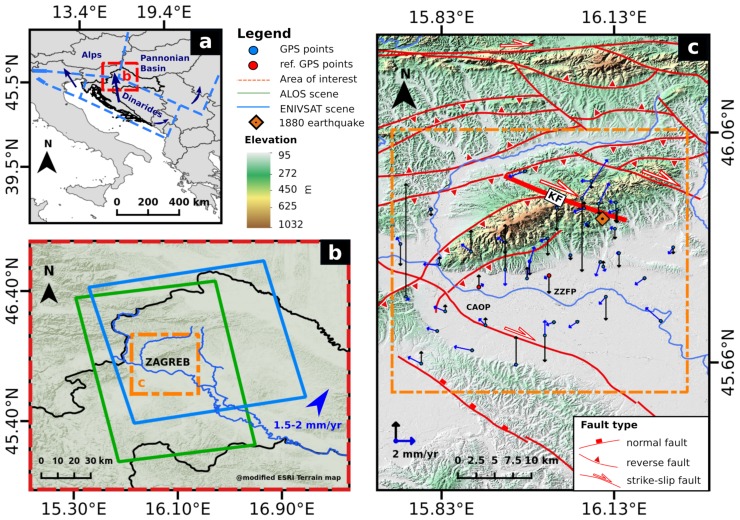
(**a**) Location map and main tectonic units of the study area. The blue arrows represent the relative movements between the tectonic units based on Tomljenović and Csontos [4] (**b**). Location map of the study area (yellow frame) and the Advanced Land Observing Satellite (ALOS) Phased Array L-band SAR (PALSAR) and Environmental Satellite (Envisat)-Advanced Synthetic Aperture Radar (ASAR) scene coverage. Blue triangle represents the direction of regional deformation trends (Sources: References [5,6] (**c**) Fault map of the study area (source: Reference [7]) and the epicenter of the 1880 great Zagreb earthquake (source: Reference [1]). Mt. Medvednica is shown in the center of the study area (yellow frame). The map also presents the locations of Global Positioning System (GPS) stations (black circles) installed by the project “The GPS network of the City of Zagreb.” Horizontal and vertical movements on GPS stations are represented with blue and black arrows, respectively (Sources: [8,9]).

On 9 November 1880, a strong earthquake (estimated M_*L*_ ≈ 6.3 reported in Prelogović et al. [1]) hit the wider Zagreb area and caused destruction of 13% of buildings in the city of Zagreb. Heavy damage was reported in nearby settlements North-East of the city, where almost all masonry buildings were destroyed in the villages of Planina, Čučerje, Vugrovec and Kašina. The area afterward experienced two more earthquakes M_*L*_ ≈ 5.9 in 1905 and 1906 with epicenters that coincide with the 1880 earthquake [2]. The earthquake epicenters were estimated to be on the Southern segment of the strike-slip Kašina Fault, that crosscuts Mt. Medvednica (Figure 1c). Based on seismological data of the past 100 years, a 150 years recurrence interval for earthquakes magnitude M_*L*_ > 6 is estimated for the wider Zagreb area (see Markušić and Herak [3]), suggesting that the next strong earthquake might occur in the next decades. Nowadays, the wider Zagreb area has significant socio-economic importance for the Republic of Croatia as it generates over 50% of the Croatian gross national product and home for about 30% of the nation’s population. Hence, there is a necessity to assess the seismic hazard in the area by investigating the ongoing geodynamic and interseismic strain accumulation processes that could evoke a next strong earthquake.

In the absence of earthquake events above magnitude M_*L*_ 5 since 1933 [3], geodetic studies were used to measure and track interseismic related ground deformations across the wider Zagreb area. In 1997, the geodetic-geodynamic project “The Geodynamic GPS Network of the City of Zagreb” was established to quantify and characterize ongoing ground deformation in the area. The project included the construction of a geodynamic network consisting of 43 specially stabilized Global Positioning System (GPS) monuments distributed across the wider Zagreb area. Since 1997, eight GPS campaigns were conducted (1997, 2001, 2004, 2006, 2007, 2008, 2009 and 2015) on the network (41 points from 2001). The GPS results revealed average horizontal movements of 1.3 mm/year and average vertical movements of 3.5 mm/year for the period 1997–2008 [8]. The GPS results indicate above-average displacements of >2 mm/year on stations around the Kašina Fault and in overall an N-S compression across Mt. Medvednica (see more details in References [8,9]).

Satellite radar interferometry (InSAR) was introduced into the project in 2015 (Pribičević et al. [10]) to improve GPS findings with high spatial resolution ground deformation analysis. The authors applied multi-temporal, Persistent Scatterers InSAR (PSI) technique performed on 40 Envisat-ASAR images (23 descending and 17 ascending tracks) covering the 2004–2009 period. Obtained results revealed mean “line-of-sight” ground velocities in the range of −4 to 4 mm/year from the ascending track and in the range of −2 to 2 mm/year for the descending track. The above-average surface velocity values were detected in the hilly part of Mt. Medvednica, inferring 1.5 mm/year uplift. The Pribičević et al. [10] presented preliminary PSI results over the study area with no detailed analysis and connection to interseismic strain accumulation in the area, which together with an insufficient data coverage of the area near the Kašina Fault, left some unanswered questions regarding the seismic hazard assessment for the wider Zagreb area.

In this study, we focus on providing an initial surface deformation analysis of interseismic strain accumulation on the Kašina Fault based on InSAR data. We were able to achieve that by analyzing two ascending InSAR datasets, 14 Advanced Land Observing Satellite (ALOS) Phased Array L-band SAR (PALSAR) images covering the period 2007–2010 and 27 Environmental Satellite (Envisat)-Advanced Synthetic Aperture Radar (ASAR) images covering the period 2002–2010, using the multi-temporal Small Baseline InSAR (SBI) data processing approach [11] together with additional tropospheric phase correction [12]. The main idea was to exploit better performance of SBI over PSI methods in rural areas and to benefit from longer wavelength L-band (ALOS-PALSAR) higher interferometric coherence over vegetated areas to achieve a better spatial coverage of the Kašina fault. The SBI results combined with GPS measurements of the study area are then used to investigate interseismic strain accumulation on the Kašina Fault in accordance to assess seismic hazard for the wider Zagreb area.

## 2. Study Area

The wider Zagreb area is located in the NW part of Croatia, centered around Croatia’s capital city (Zagreb), at the junction of three tectonic active units Dinarides, Alps and Pannonian Basin (Figure 1a). Geodynamic models of the area suggest that the Dinaridic unit moves North-West towards the Southern East-West oriented border of the relatively stable Alpine tectonic block and right-laterally with respect to the Pannonian tectonic unit [4] (Figure 1a). GPS results from the studies ([5,6] indicate northeastward regional crustal movements in the range of 1.5–2 mm/year (blue triangle in Figure 1b)). Prominent recent geodynamic activity, corroborated by historical and instrumentally recorded seismicity in the area, often evokes a significant number of moderate to strong earthquakes. In recent history, the most significant earthquakes occurred in 1775 (VII-VIII on Mercalli-Cancani-Sieberg scale (MCS°)), 1880 (VIII MCS°), 1905 (VII-VIII MCS°) and 1906 (VII-VIII MCS°) [2]. These earthquakes caused devastating effects on the city of Zagreb and nearby settlements, especially the “Great Zagreb earthquake” in 1880. Earthquake epicenters were documented to be in the area 17 km North-East of the city around Planina village (Figure 1c, shown as an orange diamond marker), located on the Southern hills of Mt. Medvednica [2]. According to Prelogović et al. [1], the Great Zagreb earthquake of 1880 occurred on a Southern segment of the Kašina Fault (KF), characterized as NW-SE trending right-lateral strike-slip fault (Figure 1c). Seismic activity in the area continues to this day but with an instrumentally recorded activity that rarely exceeds M_*L*_ 4 [13,14,15,16,17]. Considering the ongoing seismicity in the study area, our research is primarily focused on interseismic surface deformation analysis over the Kašina Fault.

## 3. Small Baseline Interferometry

Multi-temporal InSAR (MT-InSAR) techniques: Persistent Scatterers (e.g., Ferretti et al. [18]) and Small Baseline (e.g., Berardino et al., Lanari et al., Hoopper, Hetland et al. [11,19,20,21]) were developed to overcome limitations of the conventional InSAR, mostly due to decorrelation and to compensate phase erroneous contributions due to atmospheric phase delay, inaccurate topographic model and uncertain satellite orbits. Small Baseline Interferometry (SBI) processing technique focuses on the exploitation of temporal phase behavior of distributed scatterers to generate time series and overcome the sources of phase error. Distributed scatterers can be found in the radar image resolution cell where all scatterers have a comparable reflection response, usually find in rural areas and the obtained phase is produced as a sum of their random phase contribution. Thus, making them prone to temporal and spatial decorrelation in the interferogram generation. By a combination of multiple interferograms generated with small temporal and spatial baselines, a large number of resolution cells with distributed scatterers can preserve a coherent interferometric phase and be used in time series analysis. The results are relative one-dimensional “line-of-sight” (LOS) surface movements with respect to satellite imaging geometry, where 95% of LOS movement is associated with vertical and East-West ground movements. Comparison of four different SBI approaches by Gong et al. [22], shown that StaMPS (Stanford Method for Persistent Scatterers)- Small Baseline (SB) approach provides the best results in areas covered by forest and scrubs, which is the closest approximation to the terrain characteristics around the Kašina Fault. Therefore, we apply StaMPS-SB [20,23] technique on two different datasets; ALOS-PALSAR images and Envisat-ASAR images covering the period 2007–2010 and 2002–2010, respectively.

### 3.1. Data

The study area was analyzed with C-band and L-band data covering the period 2002–2010. We used 14 ascending ALOS-PALSAR satellite images covering a period from February 2007 to December 2010 and 27 ascending Envisat-ASAR satellite images covering a period from December 2002 to July 2010 (Figure 1b). Both ALOS-PALSAR’s fine modes: single-polarization (FBS) and dual-polarization (FBD) HH (Horizontal-Horizontal) images were used in the study. The advantage of using L-band (wavelength 24 cm) data is in its ability to preserve high interferometric coherence over heavily vegetated areas, which can be found over Mt. Medvednica, a near-field zone around the Kašina Fault [24]. The C-band (wavelength 5.6 cm) Envisat-ASAR images were used to ensure a higher precision of ground movements in the area, due to smaller wavelength observations and a longer time span of observations. We used the ascending Envisat-ASAR interferometric stack updated from the previous study of Pribičević et al. [10] by expanding the investigation time-window with 10 additional images.

The combination of both datasets was used to better assess surface deformation in the study area, with a primary focus on the Kašina Fault. Details concerning the used satellite missions in SBI processing can be found in Table 1.

We used an external digital elevation model (DEM) to minimize topographic phase errors in the interferogram generation. High spatial resolution (3.5 m × 3.5 m) Croatian Digital Terrain Model (DGU DTM) was used as an external DTM for topographic phase corrections in the processing. The DGU DTM was downsampled to a 15 m spatial grid to align with the size of SAR image resolution cell. We also used ERA5 reanalysis climate model [25] in the estimation and removal of stratified tropospheric effect in SBI processing.

The GPS results covering the period 1997–2015 were provided by Pribičević et al. [26] and used in the interseismic strain accumulation analysis over the Kašina Fault. The GPS data were processed in series according to conducted GPS campaigns: 1997–2001, 2001–2004, 2004–2006, 2006–2007, 2007–2008, 2008–2009 and 2009–2015, which were then combined in a single solution by using GAMIT–GLOBK software package [27]. The long term horizontal and vertical velocities on 39 GPS sites were obtained through a network adjustment with respect to two network reference points; CAOP and ZZFP (See Figure 1c).

### 3.2. Data Processing and Post-Processing

Data processing was conducted in three steps: network selection, interferogram generation and SBI time series analysis. Our analysis also includes a post-processing step, which includes filtering of the obtained velocity field, eliminating results with high standard deviation.

**Figure 2:**
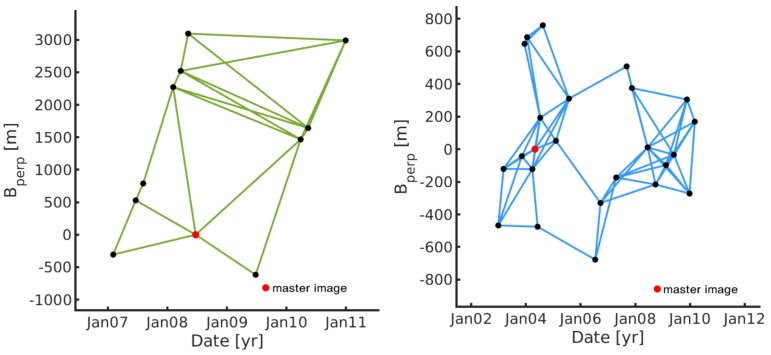
SBI networks of ALOS-PALSAR (**left**) and Envisat-ASAR (**right**) datasets.

The network selection is a necessary step for deciding which interferometric pairs will be used in SBI processing. The selection criteria for interferogram used in SBI processing is based on minimizing the perpendicular (spatial/physical separation), temporal (separation in time) and Doppler (difference between Doppler centroids) baselines between two satellite SAR acquisitions. We selected interferometric pairs using a perpendicular baseline threshold of 30% of the critical baselines (Table 1) to form an optimal SBI network, which is 1830 m and 330 m for ALOS-PALSAR and Envisat-ASAR, respectively. In this study, the selection of interferometric pairs had to be additionally readjusted to assure a fully connected SBI network, which is the requirement for 3D unwrapping in a spatial and temporal domain with SBI technique in StaMPS toolbox. Networks used in SBI processing of ALOS-PALSAR and Envisat-ASAR datasets are depicted in Figure 2.

In interferogram generation stage, we applied precise orbit information to SAR images (Delft Orbital Data Records from DEOS [28] for Envisat-ASAR and JAXA auxiliary file for ALOS-PALSAR), for coarse registration of SAR acquisitions and removal of flat reference phase contributions. Topographic phase contributions were corrected by using the DGU digital terrain model. Afterward, the corrected interferograms were filtered using spectral shift compensation in range and azimuth direction and geocoded to be used in SBI processing. Theoretical background on interferogram formation procedure, corrections and additional steps such as filtering and geocoding are described in Hanssen [29]. Coregistration and generation of interferograms were conducted using the Delft Object-oriented Radar Interferometric Software (DORIS) package developed at the Delft University of Technology for InSAR processing [30].

SBI processing was done in Stanford Method for Persistent Scattererrs/Multi-Temporal Interferometry (StaMPS-MTI) software toolbox originally developed at Stanford University, upgraded at University of Iceland and Delft University of Technology and currently under development at University of Leeds [20,23,31,32]. We applied additional orbital (a planar phase removal), topographic (removal of spatially-correlated look angle phase) and tropospheric phase contributions through SBI processing. We used the StaMPS complementary TRAIN (Toolbox for Reduction Atmospheric InSAR noise) software package [12] to estimate stratified tropospheric noise in each interferogram based on an auxiliary data from ERA5 atmospheric reanalysis model. We also applied the oscillator drift correction in the time domain according to Marinkovic et al. [33] in processing of Envisat-ASAR data, whereas we discarded 14 interferometric pairs from ALOS-PALSAR stack due to observed strong ionospheric phase noise contamination. Input parameters used for SBI processing are presented in Table 2.

ALOS-PALSAR and Envisat-ASAR datasets were processed using StaMPS-SB time series module separately due to differences in imaging geometry and wavelength. We applied a lower threshold for identification of noisy distributed scatterers (DS) points (weed standard deviation) than a default value (1), which resulted in a more reliable velocity model but with less DS points. We also had to adjust the unwrapping process to a grid size of 1000 m and a time-window of 1500 days to suppress random phase jumps in space and time domain that do not fit the expected long-wavelength tectonic signal. Processing of both datasets was done with respect to the same reference pixel, collocated with the GPS station Zavod za fotogrametriju/Institute for Photogrammetry Zagreb, Croatia (ZZFP) used as one of references in GPS processing.

A post-processing step of velocity filtering was performed on the obtained relative surface velocities and their standard deviations to exclude outliers caused by isolated unwrapping errors in SBI processing. We remove all velocities with standard deviation higher than two sigma. A total number of removed DS points are 2693 in ALOS-PALSAR velocity model and 828 DS points in Envisat-ASAR velocity model. The statistical description of results before and after filtering are shown in Table 3.

## 4. Results

### 4.1. Small Baseline Interferometry Velocity Fields

**Figure 3:**
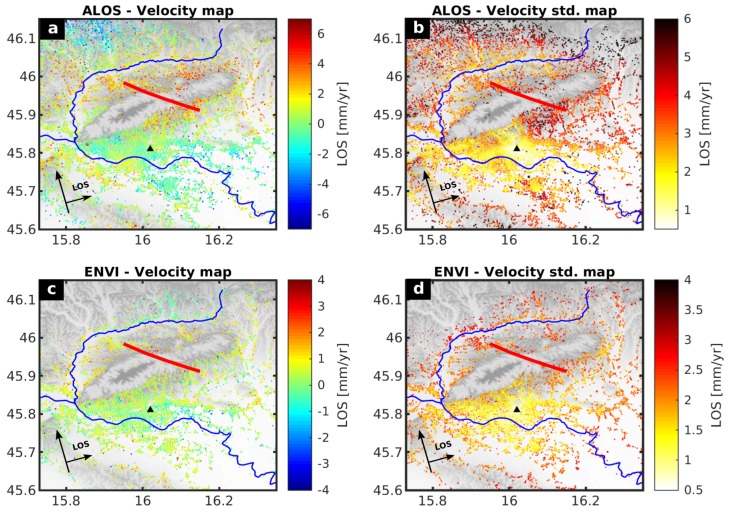
Small baseline interferometry (SBI) results for the wider Zagreb area with the reference to the black triangle and with the Kašina Fault location (red line). The blue lines mark the Sava and Krapina rivers. (**a**) ALOS-PALSAR relative velocities estimated for 2007–2010 period (**b**) Standard deviation of ALOS-PALSAR velocity values, (**c**) Envisat-ASAR relative velocities estimated for 2002–2010 period, (**d**) Standard deviation of Envisat-ASAR velocity values.

The SBI results for the wider Zagreb area Figure 3 show relative surface velocity values that represent movements in LOS direction regarding imaging geometry and satellite position with respect to a “stable” reference point (Black triangle in Figure 3). The results are presented in LOS, in which positive values (red) are towards and negative (blue) away from the satellite. Thus, positive velocities could be associated with uplift or westward ground movements and negative velocities with subsidence or eastward ground movements.

The ALOS-PALSAR velocity field (Figure 3a) shows a pattern of mean velocity increase of 1.7 mm/year centered around Mt. Medvednica, with the highest value of 2.3 ± 1.4 mm/year located at the Northern margin of Mt. Medvednica hills. ALOS-PALSAR velocities uncertainty (Figure 3b) increases from ≈1.8 mm/year over 20 km to ≈5.0 over 30 km distance from the reference point. We also observed more than 2σ velocity standard deviation in the ALOS-PALSAR velocity field in the area 8 km northeast from the reference point. At the same time, the Envisat-ASAR velocity map (Figure 3c) shows velocity increase of 0.8 ± 0.7 mm/year on the eastern part of Mt. Medvednica relative to the reference point South-West of it, which could represent the strain accumulation along the Kašina Fault. We also observed a velocity increase of 0.9 ± 0.7 mm/year on the NW foothills of Mt. Medvednica, similar as in the ALOS-PALSAR velocity field. Envisat-ASAR velocity uncertainties increase from ≈1.2 mm/year over 10 km to ≈2.1 mm/year over 20 km distance from the reference point. (Figure 3d). Both velocity maps indicate the LOS velocity increase ≈1.1 mm/year on the Northern foothills of Mt. Medvednica, which could be indicative of vertical ground movements associated with existing reverse faults (Figure 3c).

### 4.2. Interseismic Strain Accumulation Analysis on the Kašina Fault

**Figure 4:**
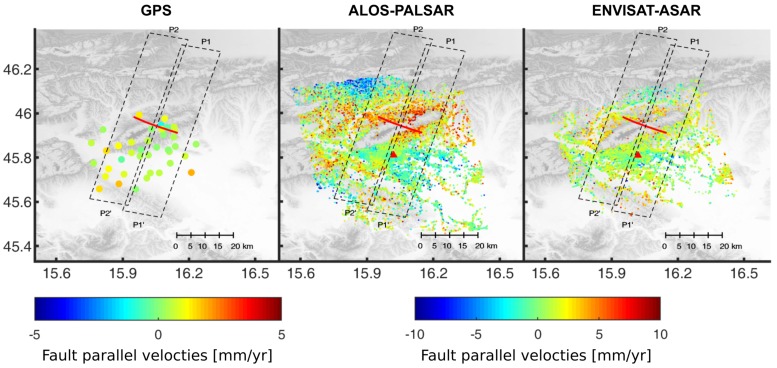
Relative GPS and SBI fault-parallel velocity fields with the location of fault-perpendicular transects (P1’-P1 and P2’-P2) over the Kašina Fault (red line) analyzed in Figure 5. The dashed lines represent the area covered by each transect. The reference point for SBI velocity maps (ALOS-PALSAR and Envisat-ASAR) is marked with a red triangle.

Previous studies have shown that the Kašina Fault is a right-lateral strike-slip fault ([1,4,34]). Thus, with the assumption that the vertical motion on the fault is minimal, around zero, we projected LOS SBI and GPS velocities in the horizontal fault parallel movements V_*fault*_ (Figure 4) by taking into account γ, the local fault strike at N70°W:

$$\begin{array}{cc} V_{fault}= & V_{SBI}/\left(-\sin\gamma \cdot \cos\alpha \cdot \sin\theta +\cos\gamma \cdot \sin\alpha \cdot \sin\theta \right)\\ V_{fault}= & H_{v_{GPS}}\cdot \cos\left(\beta -\gamma \right) \end{array}$$

where V_*SBI*_ are SBI line-of-sight velocities, θ is radar incidence angle, α is SAR satellite azimuth, $H_{v_{GPS}}$ is GPS horizontal movement and β is the azimuth of GPS horizontal movement. Results in Figure 4 indicate a mean small right lateral movements ≈ 1.7 mm/year in Envisat-ASAR and ALOS-PALSAR fault-parallel velocity maps. In contrast, GPS fault-parallel velocities are inconsistent with the expected right-lateral horizontal movements on the Kašina Fault, which could be due to the sparse distribution of GPS stations in the NE part of the study area.

**Figure 5:**
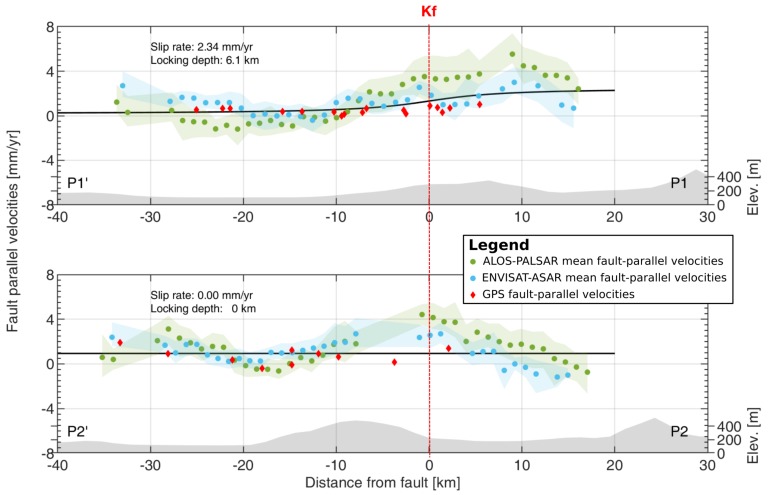
Mean rate line-of-sight (LOS) and fault-parallel velocities along on 2 normal (P1, P2) transects with respect to the Kašina Fault (red vertical dashed lines). Green and blue circles are calculated mean velocities with standard deviation along transects for ALOS-PALSAR and Envisat-ASAR data, respectively. Red diamonds mark GPS fault-parallel velocities, whereas black lines are the best fitted first-order simple elastic dislocation models. The shaded gray area represents mean terrain elevation values along transects.

We analyzed the fault-parallel movements along 2 normal transects 80 km long to investigate fault-parallel motions indicative of possible interseismic strain accumulation on the fault. We choose to analyze movement rates on two fault segments each length 14 km. The reason lies in the fact that Southern Kašina Fault segment had strong seismic activity in period 1880–1906, while Northern Kašina Fault segment appears to lack any moderate seismic activity (see Herak et al. [2]). Velocities were analyzed 40 km before and 30 km after the Southern segment (P1’-P1) and Northern segment (P2’-P2) of the Kašina Fault. We calculated the weighted mean LOS fault-parallel rate of all points on transect within 7 km in 1 km bins, selected to provide an appropriate point density along transects. Results in Figure 5 pinpoint to a small gradient of 0.2 mm/year per 1 km starting 20 km before the fault on transect P1’-P1 in Envisat-ASAR and ALOS-PALSAR fault-parallel velocity field. GPS fault-parallel velocities also show a small gradient of 0.1 mm/year per 1 km starting 20 km before the fault on both transects. Both SBI fault-parallel velocity fields show a negative gradient of 0.2 mm/year per 1 km starting 10 km before the fault on the transect P2’-P2 (Figure 1c). The uncertainties of >2.5 mm/year apply to both transect lengths from −40 to−20 km and −5 to 20 km for ALOS-PALSAR velocities. The mean uncertainty of 1.6 mm/year on transects can be found in Envisat-ASAR velocities.

Furthermore, we modeled interseismic strain accumulation on the fault by fitting a simple first-order interseismic arctangent dislocation model (see Savage and Burford [35] for details) to fault-parallel velocities along the transects. The model assumes that the dislocation occurs between the locked part of the fault plane (shallow upper crust) at the given depth (the locking depth) and lower sliding part of fault plane extending to infinity. According to the dislocation model, the fault-parallel velocities due to slip along fault *v*(*x*) can be expressed as:

(1) $$v\left(x\right)=\frac{s}{\pi }\cdot \mathrm{atan}\left(\frac{x}{D}\right)+\alpha$$

where s is the deep fault slip rate, *D* the locking depth, *x* is the distance from the fault and α is an offset. We excluded ALOS-PALSAR SBI mean velocities with more than 2σ standard deviation along transect. The best-fitting dislocation model was obtained by a weighted non-linear least-square minimization. Velocity variance of each bin along transect was used as weight in the inversion. We obtained an slip rate estimate of 2.3 mm/year with locking depth of 6.1 km on Southern segment of the Kašina Fault (transect P1’-P1, see Figure 5). However, we could not obtain any slip rate estimate on the Northern Segment (transect P2’-P2, see Figure 5).

## 5. Discussion

This study is focused on the characterization of possible interseismic deformation along the Kašina Fault to provide seismic hazard assessment for the wider Zagreb area. The relative shortening of the distance between the satellite and the ground can be found on North-East part with the respect to the South-West part of Mt. Medvednica by 0.5–1.0 mm/year, which corresponds with the location of the Kašina Fault and could be indicative of strain accumulation. This LOS velocity change is consistent with ≈1.2 mm/year LOS velocity change in previous published PSI velocity fields [10]. Nevertheless, ALOS-PALSAR velocities should be taken with caution due to a high level of velocity uncertainty observed in the ALOS-PALSAR velocity standard deviation map. The explanation could be in less than an ideal number of available ALOS-PALSAR images for the SBI processing [23].

The combined GPS and SBI velocities were used in the analysis of interseismic strain accumulation on two fault-perpendicular transects along Kašina Fault. Our modeling shows a potential ongoing shearing on the Southern segment of Kašina Fault with an estimated slip rate of 2.3 mm/year at locking depth of 6.1 km. This rate corresponds to the geological rate of 2.5 mm/year reported by Kuk et al. [36]. On the other hand, we could not find any geodetic evidence for slip rate on the Northern segment of the Kašina fault. Possible explanation is that the slip rate is currently below the reported SBI measurement uncertainty level of 1.5 mm/year [37] on the Northern segment. The transect P2’-P2 (see Figure 4) also covers the active SE dipping reverse fault North Medvednica Boundary fault (see Figure 1c), which motion (see Matoš et al. [7] for the details) could also bias the estimation of the slip rate.

The estimated slip rate and locking depth are used to obtain a first-order geodetic estimate of the seismic potential of the Southern segment of the Kašina Fault. We use a rigidity of 30 GPa for the conversion from slip to seismic moment. For this 14 km long segment, the tectonic loading rate of 2.3 mm/year corresponds to 0.26 m slip accumulated over the 113 years since the last strong earthquake in 1906. By using a locking depth of 6.1 km, the total slip rate over the time implies that the Southern segment of the Kašina Fault has the potential to generate earthquake magnitude M_*w*_ ≈ 5.86. This explains the lack of moderate to strong earthquakes in the past 100 years and corresponds well with earthquake magnitude M > 6 recurrence interval of 150 years [3]. Nevertheless, given the model assumptions (2-D geometry), lack of the GPS coverage of NE side of the fault and level of uncertainty in SBI data, we recognize that the interseismic slip rate estimate of the Kašina Fault should be taken as a preliminary result.

We expect a potential improvement in analyzing interseismic slip rate on the Kašina fault with a InSAR data acquired by the new SAR satellites such as Sentinel-1A/B. Covering the same time span of 8 years as the used Envisat-ASAR data in this study but with more systematic and frequent acquisitions (more images per month) should result in a lower velocity uncertainty level and more reliable slip rate estimate on the fault. We also see a further improvement of the research in the installation of corner reflectors for SAR observations around the fault, which would ensure stable and reliable monitoring points in this highly vegetated area. We strongly suggest an expansion of GPS network in the NE part of the study area to obtain better spatial coverage of the fault, with the preferable introduction of continuous GPS observations. It would be also important to conduct a paleoseismic trenching to obtain a seismotectonic reference data in terms of strong earthquake recurrence interval on the fault. Nevertheless, it is important to point out that the expected progress was achieved in terms of an initial geodetic investigation of interseismic surface deformation on the Kašina Fault.

## 6. Conclusions

The SBI results of this study indicate very small ground surface deformations within ±3.5 mm/year in the study area. The tectonic signal is observed as a relative “line-of-sight” velocity change of ≈1 mm/year on NE part of Mt. Medvednica, consistent with the previous PSI study. Almost double velocity uncertainties in ALOS-PALSAR velocity field are presumably due to the less than ideal size of dataset used in the analysis. Preliminary interseismic strain analysis on the Kašina Fault suggests that the Southern segment of the fault has a potential to generate earthquake magnitude M_*w*_ < 6, whereas there is no geodetic evidence for strain accumulation on the Northern segment. The location of detected strain accumulation on the fault corresponds with documented strong seismic activity during 1880–1906 and implies the potential seismic hazard in the study area. Further improvements in the research, should be focused on the investigation of the fault Northern segment activity with more GPS stations installed on the NE part and application of new SAR satellite missions that could provide a better measurement precision and a potential improvement in estimation of interseismic slip rate on the fault.

## Figures and Tables

**Table 1:** Satellite data used in the processing.

Satellite Mission	ALOS-PALSAR		Envisat-ASAR
**Wavelength [cm]**	23.6		5.6
**First date (yyyy-mm-dd)**	2007-02-03		2002-12-28
**Last date (yyyy-mm-dd)**	2010-12-30		2010-07-24
**Orbit direction**	Ascending		Ascending
**Inc. angle [deg]**	38.75		21.1
**Acquist. mode**	Fine Single Beam	Fine Dual Beam	Stripmap
**Imaging mode**	HH or VV	HH + HV or VV + VH	HH or VV
**Spatial resolution (range × azimuth [m] )**	4.68 × 3.13	9.37 × 3.14	7.81 × 4.04
**Critc. perp. baseline [km]**	13.1	6.5	1.1
**Numb. of images**	6	8	27

**Table 2:** Input parameters for Stanford Method for Persistent Scattererrs (StaMPS)-Small Baseline (SB) processing.

StaMPS-SB Parameters	ALOS-PALSAR	Envisat-ASAR
Number of interferograms	21	64
Weed standard deviation	0.6	0.8
Weed time window [days]	1100	1100
Merge resample size	300	300
Merge standard deviation	0.2	0.4
Unwrap grid size [m]	100	100
Unwrap time [days]	1500	1500
Reference point [Lon Lat] [deg]	16.02 45.81	16.02 45.81
Reference radius	500	500

**Table 3:** StaMPS-SB results.

Statistics		ALOS-PALSAR		Envisat-ASAR	
		Before Filtering	After Filtering	Before Filtering	After Filtering
Num. of DS points [#]		15,033	12,430	8922	8094
Velocity model [mm/year]	Median	0.51	0.52	0.40	0.41
	Interquartile range	2.01	1.99	0.77	0.75
Stand. dev. model [mm/year]	Median	3.49	3.12	2.00	1.93
	Interquartile range	2.56	1.72	0.77	0.75
